# Impact of Prunus Cerasus on PGR and HAS2 in Cumulus Cells and Fertility Outcome

**DOI:** 10.15171/apb.2016.010

**Published:** 2016-03-17

**Authors:** Fatemeh Namvar Vansofla, Leila Roshangar, Azadeh Montaseri, Jafar Soleimani Rad

**Affiliations:** ^1^ Department of Anatomical Sciences, Faculty of Medicine, Tabriz University of Medical Sciences, Tabriz, Iran.; ^2^ Cord Blood Stem Cell Research Center, Faculty of Medicine, Tabriz University of Medical Sciences, Tabriz, Iran.; ^3^ Department of Tissue Engineering, Faculty of Medicine, Tabriz University of Medical Sciences, Tabriz, Iran.

**Keywords:** Cumulus cells, HAS2, Infertility, PGR, Prunus cerasus

## Abstract

***Purpose:*** Cumulus cells have a critical role in normal oocyte development and fertilization. Prunus cerasus is an anthocyanin rich berry and performs strong antioxidant activity. The present study set to determine if Prunus cerasus can affect expression of HAS2 (hyaluronan synthase 2) and progesterone receptor in Cumulus cells and its consequences outcome of the in vitro fertilization.

***Methods:*** 60 female and 15 male adult mice were used for mating and IVF (in vitro fertilization). Prunus cerasus extraction was added to the diet of female mice for 30 days. Ovulation induction and oocytes collection were done as routine. The cumulus cells were dissected apart, and the expression of progesterone receptor and HAS2 was detected using RT-PCR (real-time polymerase chain reaction). Fertilization rate was evaluated by IVF. All data were analyzed using t-test.

***Results:*** Data was showed that expression of progesterone receptor and HAS2 in cumulus cells of mice that received prunus cerasus increased. Moreover, oocyte fertilization rate also increased significantly.

***Conclusion:*** Prunus cerasus as an antioxidant natural can become an important medication for improving oocyte quality and opening new opportunities for infertility treatment. It is concluded that Prunus cerasus consumption could improve fertility rate by increasing progesterone receptor and HAS2 activity in cumulus cells.

## Introduction

Infertility is defined as inability to obtain fertility after one year of unprotected regular intercourse‏.^1-4^ It has been reported that about 10% -15% of young couples suffer from infertility. Of these, 40%-55% are due to female factors, 20%-30% are due to male factors, and 15%-17% are unexplained infertility‏.^1^ Over the years, ART (assisted reproductive technology) has been a method for infertility treatment with different causes‏.^5,6^ In order to increase the success rate of fertilization, several herbal and chemical supplemental factors have been used‏.^7^ Because of less invasiveness and costliness nature of traditional medicine, recent research has focused on herbal use‏.^8,9^‏ Anthocyanin as secondary metabolite group of flavonoids has attracted much attention.^10^ Strong antioxidant activity was the best known property of flavonoids.^11^ Antioxidant activity of anthocyanin is associated with a variety of health benefits including inflammation, cancer, atherosclerosis, and diabetes‏.^12^ Recent studies have shown that reproductive processes may be influenced by hypothalamic-pituitary-gonadal axis via scavenging free radicals‏.^13-15^ However, interest in antioxidant has recently been intensified because of their possible effect on egg quality, fertilization, and pregnancy rates‏.^16,17^ Antioxidant therapy has been attended to alleviate infertility, and dietary antioxidant has been beneficial for female reproductive disorder.^18^ Fruits including Sour cherry (Prunus cerasus), containing anthocyanin and strong antioxidant activity, have attracted much attention‏.^19^ Prunus cerasus anthocyanins have a vast range of biochemical and pharmacological effect, and have been recommended as nutritional supplement‏.^20^ Granulosa cells consist of mural and cumulus cells in preovulatory follicles. Mural cells are in the follicle wall and cumulus cell in vicinal of oocyte‏.^21^ In the midcycle, increased level of LH result in ovulation by inducing resumption of meiotic maturation, transformation of granulosa cells, and expansion of cumulus-oocyte complex. The latter, is associated with accumulation of hyaluronic acid-rich extra cellular matrix in cumulus cells‏.^22^ During the process of cumulus expansion, the most important genes involved in the formation of matrix hyaluronic are hyaluronic synthase2 (HAS2)‏.^23^ HAS2 mRNA is necessary for cumulus expansion and signaling during ovulatory response‏.^24^ Cumulus expansion facilitates the fertilization,‏ and the failure of this process can lead to fertilization problem and is associated with low potential for implantation and decline in the quality of oocyte‏.^25,26^ Paracrine factor under the influence of progesterone is required for preimplantation embryonic development,‏ but direct or indirect effect of progesterone is not clear‏.^27^ Previous studies in our laboratory showed that the presence of Progesterone receptor is essential for female fertility and ovulation control‏.^28,29^ In this study, Prunus cerasus effect on progesterone receptor expression, HAS2 in cumulus cells and the rate of in vitro fertilization are evaluated.

## Materials and Methods

### Preparation of extract 

Sour cherry were collected from the market and authenticated by a botanist (School of Pharmacy, Tabriz University of Medical Sciences). The extract, according to World Health Organization (WHO) protocol was prepared. 500g of Sour cherry was shed-dried powdered and added to 5 Li of 70°C ethanol (v/v) and left to macerate for 20 hours at room temperature. The basin was rotated slowly during this time. After‏ filtration, ethanol at low pressure at 30 °C. The samples were stored frozen at –20 °C until used.

### Study population

A total of 60 female BALB/c mice (4-5weeks) and 15 adult male BALB/c mice were obtained from the animal house of Tabriz University of Medical Sciences (TUMS). For a period of 2 weeks prior to the experiment, the mice were kept in animal room for adapting. 1.5 kg of mice food was added to 1000ml of Prunus cerasus extract and mixed together completely. The mixed diet let‏ to‏ dry in 2 days. Female mice were randomly divided into two equal groups. In the control group, each animal received diet without Prunus cerasus. In the experimental group, each animal received 25g diet with Prunus cerasus for 4 weeks. After 4 weeks for induction of ovulation 10 IU HMG/PMSG (pregnant mare serum gonadotropin) and after 48 hours, 10 IU hCG (human chorionic gonadotropin) was injected intraperitoneally.

### Oocyte collection

One day post hCG injection, the mice were sacrificed and ovaries were removed from the female mice. Mice ovaries (n=100) were obtained and placed in sterile PBS and transported to the Tissue Engineering Laboratory in two groups. The stromal tissue surrounding the ovaries was removed, and oocyte were collected under flashing method and cultured in dishes with Universal IVF culture medium (Origio-Medicult,‏ 10300060) under sterile mineral oil.

### Isolation of cumulus cells

The cumulus cells surrounding the oocyte (COCS) were separated from oocyte using hyaluronidase enzyme. Hyaluronidase enzyme causes the connection of oocyte and cumulus cells to be released and facilitates separation. Cumulus cells were transferred to another dish containing medium. PBS was added for washing and centrifuged 2 times for 5 min. After centrifuge, the pellet of cumulus cells was centrifuged for 1 h at -20 °C and then stored at -80 °C until RNA isolation.

### In vitro fertilization

Male mice were killed by cervical dislocation, and the sperm were collected from the cauda epididymis by incubating the pieces of epididymis in 37°C Co2 incubator for 20 min. The sperm sample was added to the collected oocyte in the control group and experimental case group. The fertility success rate was assessed based on the formation of embryo.

### Real time RT – PCR

RNA expression was indicated using real time RT-PCR assay to measure HAS2 and PGR mRNA. The primers, used in PCR are presented in Table 1. Internal control gene (GAPDH) was used for normalization of the result. Total RNA of HAS2 and progesterone receptor were extracted using RNeasy Micro kit from cumulus cell. The cDNA were synthesized, and primer design was done. Real time- polymerase chain reaction (RT-PCR) was performed for gene expression analysis.

### Statistical analysis

All statistical analysis was done using SPSS software version 22 and independent sample T-test. P values≤0.05 were considered statistically significant.

**Table 1:** process of primer for Real-time RT PCR

**Gene**	**Sequence**	**Accession number**	**Base pair**
**HAS2**	Primer F: 5’GGAGGTGTTGGGGAGATGT3’ Primer R: R: 5’GTTTCAGTAAGGCATTAGATCGA3’	NM-008216	314
**PGR**	Primer F: F: 5’CTGTGCCTTACCATGTGTGGCA3’ Primer R: 5’TTCACCATGCCCGCCAGGAT3’	NM-008829.2	389
**GAPDH**	Primer F: 5’AAGCTCATTTCCTGGTATGACAACG3’ Primer R: 5’TCTTCCTCTTGTGCTCTTGCTGG3’	NM-002046	126

## Results

### Effect of Prunus cerasus on HAS2 and PGR expression in cumulus cells

**Figure 1:**
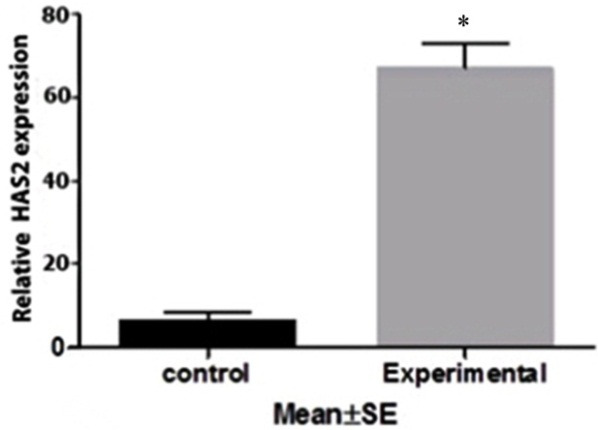
HAS2 gene expression in isolated cumulus cells in control group and mice received prunus cerasus extract * (P ≤0.0001)

**Figure 2:**
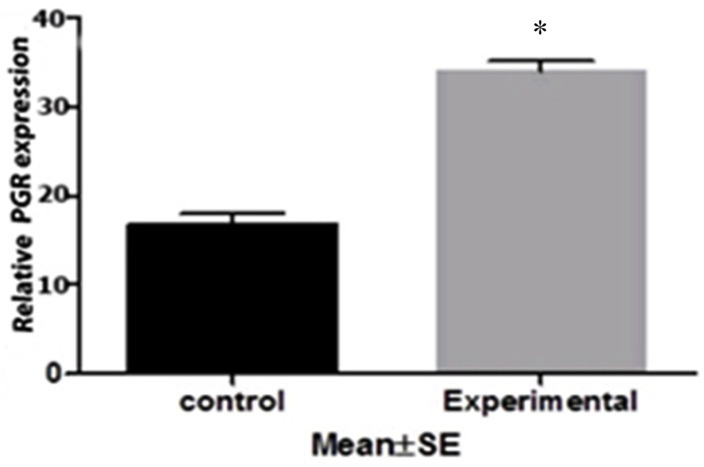
PGR gene expression in isolated cumulus cell in control group and mice received prunus cerasus extract * (P<0.05)

Expression of HAS2 and PGR mRNA in cumulus cells, isolated from mice oocyte, was assessed by quantitative real time PCR. There was a significant increase in HAS2 mRNA in the experimental group compared to the control (Figure 1). However, the expression level of PGR gene in experimental group was increased compared to the control group (Figure 2).

### Effect of prunus cerasus on fertilization rate

**Figure 3:**
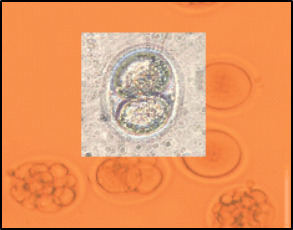
Photomicrograph of inseminated oocytes in mice received prunus cerasus extract. Note the development of several embryos.

IVF was performed in both control and experimental groups, and 100 oocytes were used per group. Counting the number of embryos revealed that in the control group which was not received Prunus cerasus,‏ 77% of oocytes were developed into embryos, while in the experimental group receiving Prunus cerasus for 4 weeks, 89% of oocytes were developed into embryos (P≤0.0001). Developed embryos in the experimental group are shown in Figure 3.

## Discussion

The results revealed that the addition of Prunus cerasus to laboratory diet resulted in increase of expression of HAS2 and PGR gene in cumulus cells and fertility success rate. Since Prunus cerasus contain significant levels of anthocyanins, strong antioxidant activity can influence fertility.^10^ Significant increase in the expression of HAS2 and PGR gene in the case group compared to the control group could be due to the loss of oxidative stress produced by antioxidant property of Prunus cerasus, which can also be justified for increasing fertility rate. Follicular fluid contains a lot of antioxidants that protect oocyte against free radicals. The disorder in pro oxidant/antioxidant system in follicular fluid can damage oocyte′s DNA, and cytoskeleton of membrane can lead to impaired fertility.^30,31^ Use of antioxidant rich food prevents progression of diseases related to oxidative stress.^32^ Free radicals that are indication of oxidative stress in many diseases are highly reactive, toxic, and short-lived molecules that hurt DNA, protein, lipid, and carbohydrates within the tissue.^32^ Oxidative stress specially affects microenvironment of oocyte, which has a detrimental influence on follicular development, ovulation, oocyte quality, implantation, and early embryonic development.^33^In the study of Damar and Eksi and other studies, anthocyanin and antioxidant capacity of Prunus cerasus is evidenced.^11,34,35^ Showell MG et al.’s study on the effects of antioxidants on fertility was contradictory, needing further investigation.^32^ Unlike the present study, Jozwick et al. did not report any relationship between free radical marker and pregnancy rate.^36^ The results of the present study is in agreement with Polak et al. who state that the concentration of antioxidant in peritoneal fluid of patients with unexplained infertility was significantly lower than the its concentration in fertile patients.^37^ Study of Agawarl et al is in line with the results of current study demonstrating that pregnancy rates in patients undergoing IVF, using antioxidant-supplemented media, were higher than standard media without antioxidants.^32^ Since studies have shown that antioxidants affect oocyte quality, high degree of expansion of cumulus cells is directly related to oocyte quality and expression of HAS2 and PGR gene is necessary for cumulus expansion. According to the results it could be postulated, it is antioxidant capacity of prunus cerasus that resulted in increased gene expression and fertilization rate.

## Conclusion

It is concluded that Prunus cerasus consumption improves fertility rate by increasing progesterone receptor and HAS2 activity in cumulus cells.

## Acknowledgments

This article is resulted from a research proposal leading to the thesis of Fatemeh Namvar, MSc student of anatomical sciences and was approved by research deputy of Tabriz University of Medical Sciences, Tabriz, Iran**.**

## Ethical Issues

Not applicable.

## Conflict of Interest

The authors declare no conflict of interest
